# Spondylolysis among Patients Without Low Back Pain in a Diagnostic Centre: A Descriptive Cross-sectional Study

**DOI:** 10.31729/jnma.8022

**Published:** 2023-02-28

**Authors:** Bishwanath Sharma, Om Biju Panta, Bikash Raj Thapa, Suresh Thapa

**Affiliations:** 1 Department of Radiology, Bheri Hospital, Banke, Nepalgunj, Nepal; 2 Department of Radiology, Nepal Mediciti Hospital, Nakhkhu Patan, Lalitpur, Nepal; 3 Department or Radiology, National Trauma Centre, Mahankal Marg, Kathmandu, Nepal; 4 Department of Radiology, Lumbini Province Hospital, Butwal, Rupandehi, Nepal

**Keywords:** *low back pain*, *spondylolisthesis*, *spondylolysis*

## Abstract

**Introduction:**

Spondylolysis can either be asymptomatic or can cause significant low back pain. It is sometimes associated with the translation of one vertebra over another and is termed spondylolisthesis. The aim of the study was to find out the prevalence of spondylolysis among patients without low back pain in a diagnostic centre.

**Methods:**

A descriptive cross-sectional study was carried out in a referral diagnostic centre from 15 December 2018 to 14 December 2021 . Ethical approval was obtained from the Nepal Health Research Council (Reference number: 2903). Images of a computed tomography scan of the abdomen performed for other abdominal causes and without low back pain were reconstructed in the sagittal and coronal plane and evaluated for the presence of spondylolysis and spondylolisthesis in the lumbar spine. Demographic data were taken from the hospital records. Convenience sampling method was used. Point estimate and 95% Confidence Interval were calculated.

**Results:**

Among 768 patients without low back pain, spondylolysis was found in 59 (7.68%) (5.809.56, 95% Confidence Interval). Spondylolisthesis was found in only 16 (27.1%) individuals with spondylolysis. The majority of spondylolysis cases were encountered in L5 level in 54 (91.53%). The mean age of patients with spondylolysis was 41.9±14.46 years. Male to female ratio was 1:1.18.

**Conclusions:**

The prevalence of spondylolysis in our study was found to be similar to other studies done in similar settings.

## INTRODUCTION

Spondylolysis is a defect in the pars interarticularis of the vertebral arch. Spondylolysis can either be asymptomatic or can cause significant low back pain.^1^ Spondylolisthesis is the forward displacement of the superior vertebra on its adjacent caudal vertebra.^1,2^ Spondylolisthesis can be related to degeneration or spondylolysis.^3-5^

Most of the previous studies have evaluated the prevalence of spondylolysis in symptomatic patients. Most of the previous studies have been conducted using plain radiographs.^6^ However, computed tomography (CT) is considered to represent a particularly accurate tool for evaluating pars defects as it can often reveal a pars fracture early without the development of spondylolisthesis.^7,8^ There is a relative paucity of data on the prevalence of this spondylolysis and spondylolisthesis in asymptomatic patients in Nepal.

The aim of the study was to find out the prevalence of spondylolysis among patients without low back pain in a diagnostic centre.

## METHODS

This descriptive cross-sectional study was done at Jeevan Raksha Hitech Diagnostic Centre, Nepalgunj. All CT scans of the abdomen done from 15 December 2018 to 14 December 2021 were included in the study. Ethical approval was taken from the Nepal Health Research Council (Reference number: 2903). Patients of all ages who underwent Computed Tomography abdomen, CT of Kidneys, Ureters and Bladder (CT KUB) / Intravenous urogram (IVU) for abdominal, pelvic and urological conditions were included in this study. Patients with a recent history of trauma, known cases of orthopaedic, neurological and degenerative spine diseases, and pathological involvement of the spine evident in CT scan (to avoid any bias) were excluded from this study. Convenience sampling method was used. The sample size was calculated using the following formula:


$$\begin{array}{ll} \text{n} & ={\text{Z}}^{2}\times \frac{\text{p}\times \text{q}}{{\text{e}}^{\text{2}}}\\ & =1.96^{2}\times \frac{0.0602\times 0.9398}{0.02^{2}}\\ & =544 \end{array}$$

Where,

Z = 1.96 at 95% Confidence Interval (CI)
p = prevalence of spondylolysis taken from previous study, 6.02%^9^
q = 1-p
e = margin of error, 2%

The minimum sample size calculated was 544. However, the final sample size taken was 768.

Images of CT scans of the abdomen in patients who underwent CT evaluation for cause unrelated to low back pain were reviewed by a single radiologist with 9 years of experience in radiology reporting. Images were evaluated in axial, sagittal and coronal reconstructions. Spondylolysis in the lumbar vertebra i.e., a defect extending through the pars interarticularis, was identified. CT scan of patients with spondylolysis was then reviewed for the presence of spondylolisthesis defined as the abnormal anterior translation of a vertebral body over another. Grading of spondylolisthesis was estimated using the Meyerding classification: Grade 0, No slip; Grade I, > 5% and < 25%; Grade II, 26-50%; Grade III, 51-75%; Grade IV, 76-100%; and Grade V, complete slippage.^10^ The level of spondylolysis and spondylolisthesis were noted. In the presence of transitional vertebra, the level was determined by counting vertebrae taking D12 as a reference. Demographic details of the patients were collected from institutional records.

Data were entered and analysed using IBM SPSS version 23. Point estimate and 95% CI were calculated.

## RESULTS

Among 768 patients undergoing computed tomography scans, spondylolysis was found in 59 (7.68%) (5.80-9.56, 95% CI). The mean age of patients was found to be 41.9±14.46 years. The median (Interquartile range) was found to be 42 (21). Majority of spondylolysis cases were encountered in L5 level in 54 (91.53%). Among total patients with spondylolysis, spondylolisthesis was seen in only 16 (27.12%) cases. Among 16 patients with spondylolisthesis, Meyerding grade I spondylolisthesis was observed in 9 (56.25%) patients whereas grade II spondylolisthesis was observed in 5 (31.25%) patients. Only 2 (12.5%) patients presented grade III spondylolisthesis. Among 16 patients with spondylolisthesis, 14 patients (87.5%) were older than 40 years of age (Table 1).

**Table 1:** Demographics of patient with spondylolysis (n= 59).

Characteristics	n (%)
**Age group (years)**	
<20	3 (5.08)
20-30	14 (23.73)
30-40	11 (18.64)
40-50	13 (22.03)
50-60	13 (22.03)
>60	5 (8.47)
**Sex**	
Female	32 (54.24)
Male	27 (45.76)
**Vertebra involved in lysis**	
L4	3 (5.08)
L5	54 (91.53)
L4+L5	2 (3.39)
Spondylolisthesis	16 (27.11)
**Grade**	
I	9 (15.25)
II	5 (8.47)
III	2 (3.39)
IV	-

## DISCUSSION

The prevalence of spondylolysis was 7.68% in our study which was found to be similar to other study where spondylolysis was reported in 6.02%.^9,11^ According to these earlier studies, most cases of spondylolysis arise in early childhood, and 4.4% of children entering first grade have spondylolysis on screening plain radiographs.^9^ It has been thought that the prevalence increases to 6% by age 18 and remains stable at that rate throughout adulthood. However, another study conducted in India showed a prevalence of 12.5% spondylolysis on examining 852 consecutive patients with abdomen CT in an unselected Indian population which was significantly higher than our study.^12^

The possible explanation for the slightly higher rate identified in the current study is the use of CT scan. This imaging modality is currently considered the gold standard in terms of identifying spondylolysis, particularly in the setting of unilateral defects, and non-displaced bilateral defects. Most previous studies of spondylolysis prevalence, including the oft-cited Scandinavian population study, have reported data from large screening programs based solely on plain radiographs.^11^

In the present study, we found that females had a higher prevalence of spondylolysis as compared to males. The female-to-male ratio of almost 1:0.84 in the current study is more than the 1:2 ratio reported in other studies.^13-17^ The vast majority of spondylolysis cases involved the L5 vertebral level (91.5%) in our study. Another study showed the incidence of spondylolysis at L5 vertebral level to be (85-95%) and L4 level (5-15%).^1^ Similarly, in our study, higher incidence was found at L5 level. In other studies, the incidence of multiple lumbar spondylolysis appears to vary between 1.2% to 5.6%.^11,18,19^ In our study, multilevel spondylolysis was seen in 1.8%.

The presence of spondylolysis in 72.89% without any measurable spondylolisthesis in our study is noteworthy and reinforces the point that these lesions may easily be missed by standard plain radiographic evaluation. Other authors have reported a different finding: up to 55% of patients diagnosed with spondylolysis did not progress to spondylolisthesis.^18-20^

This was not population-based study and was a retrospective hospital record-based study which might not give a true representation of the population.

## CONCLUSIONS

The prevalence of spondylolysis in our study was similar to other studies done in similar settings.

## References

[1] Syrmou E, Tsitsopoulos PP, Marinopoulos D, Tsonidis C, Anagnostopoulos I, Tsitsopoulos PD (2010). Spondylolysis: a review and reappraisal.. Hippokratia..

[2] Kalichman L, Kim DH, Li L, Guermazi A, Berkin V, Hunter DJ (2009). Spondylolysis and spondylolisthesis: prevalence and association with low back pain in the adult community-based population.. Spine (Phila Pa 1976)..

[3] Wiltse LL, Winter RB (1983). Terminology and measurement of spondylolisthesis.. J Bone Joint Surg Am..

[4] Fitzgerald JA, Newman PH (1976). Degenerative spondylolisthesis.. J Bone Joint Surg Br.

[5] Bookhout MR (1993). Evaluation and conservative management of spondylolisthesis.. J Back Musculoskelet Rehabil..

[6] Sonne-Holm S, Jacobsen S, Rovsing HC, Monrad H, Gebuhr P (2007). Lumbar spondylolysis: a life long dynamic condition? A cross sectional survey of 4.151 adults.. Eur Spine J..

[7] Teplick JG, Laffey PA, Berman A, Haskin ME (1986). Diagnosis and evaluation of spondylolisthesis and/or spondylolysis on axial CT.. AJNR Am J Neuroradiol..

[8] Krupski W, Majcher P, Tatara MR (2004). Computed tomorgaphy diagnostic of lumbar spondylolysis.. Ortop Traumatol Rehabil..

[9] Fredrickson BE, Baker D, McHolick WJ, Yuan HA, Lubicky JP (1984). The natural history of spondylolysis and spondylolisthesis.. J Bone Joint Surg Am..

[10] Gaillard F, Rock P, El-Feky M (2020). Spondylolisthesis grading system [Internet]..

[11] Virta L, Ronnemaa T, Osterman K, Aalto T, Laakso M (1992). Prevalence of isthmic lumbar spondylolisthesis in middle-aged subjects from eastern and western Finland.. J Clin Epidemiol..

[12] Chellathurai A, Kannappan S, Melba F, Gnanasigamani S, Balasubramaniam S (2018). Evaluation of incidental lumbar spondylolysis by computed tomography.. Journal of Evolution of Medical and Dental Sciences..

[13] Roche MA, Rowe GG (1951). The incidence of separate neural arch and coincident bone variations: a survey of 4200 skeletons.. The Anatomical Record..

[14] Wiltse LL, Widell EH, Jackson DW (1975). Fatigue fracture: the basic lesion is inthmic spondylolisthesis.. J Bone Joint Surg Am..

[15] Wiltse LL, Newman PH, Macnab I (1976). Classification of spondylolysis and spondylolisthesis.. Clin Orthop Relat Res..

[16] Newman PH (1976). Stenosis of the lumbar spine in spondylolis thesis.. Clin Orthop Relat Res..

[17] Beutler WJ, Fredrickson BE, Murtland A, Sweeney CA, Grant WD, Baker D (2003). The natural history of spondylolysis and spondylolisthesis: 45-year follow-up evaluation.. Spine (Phila Pa 1976)..

[18] Park KH, Ha JW, Kim HS, Moon ES, Moon SH, Lee HM (2009). Multiple levels of lumbar spondylolysis - a case report.. Asian Spine J..

[19] Saraste H (1993). Spondylolysis and spondylolisthesis.. Acta Orthop Scand Suppl..

[20] Stewart TD (1953). The age incidence of neural-arch defects in Alaskan natives, considered from the standpoint of etiology. J Bone Joint Surg Am..

